# The Advantages of the X-Ray in the Treatment as Well as the Diagnosis of Certain Surgical Cases

**Published:** 1901-09

**Authors:** M. F. Carson

**Affiliations:** Griffin, Ga.


ATLANTA

Journal-Record of Medicine.

Successor to Atlanta Medical and Surgical Journal, Established 1855, and Southern Medical Record, Established 1870.

VOL. III. SEPTEMBER, 1901. No. 6.

BERNARD WOLFF, M.D., EDITOR, Nos. 319–20 Prudential.

Published Monthly.

M. B. HUTCHINS, M.D., BUSINESS MANAGER, No. 64 Marietta St.

### *ORIGINAL COMMUNICATIONS.*

### THE ADVANTAGES OF THE X-RAY IN THE TREATMENT AS WELL AS THE DIAGNOSIS OF CERTAIN SURGICAL CASES.*

BY M. F. CARSON, M.D.,
GRIFFIN, GA.

One of the most interesting features of medicine and surgery in this, the beginning of the new century, is the increasing interest in X-ray work, which is perhaps accounted for by a large number of our physicians and surgeons, remaining indifferent for so long to this important subject, beginning to appreciate its importance.

No surgeon who once having witnessed a high efficiency X-ray demonstration can fail to desire so valuable a recourse. Observations upon fractures and dislocations by the aid of the X-ray has convinced eminent authorities of the necessity of many changes in our text-books, based upon the revelations of the ray.

*Read before the meeting of the Medical Association of Georgia, at Augusta, April, 1901.

In the examination of soft parts much valuable information has been gained. In presenting this paper I do not claim anything new or original, but desire to emphasize the importance of this valuable assistant in the diagnosis and treatment of certain surgical diseases. I desire to present the following cases for the purpose of demonstrating that in the X-ray we possess an accurate agent for diagnosing tubercular changes of lung tissue in its various stages. My first case, male, age twenty-four, for several weeks had been losing flesh, accompanied by severe cough. Examination showed dullness of the upper lobe of the right lung with crepitant and subcrepitant rales. This patient expectorated freely very foul sputa which under the microscope showed pus in abundance. X-ray examination showed a distinct circumscribed area as large as an orange. Before a large tube glowing at its maximum of intensity, I introduced a long needle attached to an ordinary syringe guided by the X-ray, and withdrew considerable pus. Remembering Dr. Seneca D. Powell's address before the Southern Surgical Association last year on how he used pure carbolic acid in abscesses, I resolved to try it in this case. I injected into the center of this dark area sixty minims of pure carbolic acid, followed in five minutes with double the quantity of pure alcohol. This operation I repeated every day for five days, withdrawing considerable pus each time. On the thirtieth day I examined this patient before the fluoroscope to find this lung perfectly clear. I placed this patient on iron and cod liver oil. His cough has subsided and he has gained steadily. I have frequently examined this patient with the ray to find this once dark area perfectly clear. Physical signs show to-day a distinct cavity.

This you may consider heroic treatment. But I believe that by the injection of carbolic acid I produced a cavity and at least arrested the progress of a tubercular abscess. I consider it just as safe to inject pure carbolic acid into a tubercular lung as in a tubercular abscess elsewhere, especially when we can regulate the degree of its effect by the use of alcohol.

I have frequently demonstrated the dark shadows in the incipient stage of tuberculosis, where the physical signs were negative.

While much has been written and claimed for the X-ray lately as a theraputic agent in the treatment of tubercular disease of the lungs, skin and elsewhere, after a thorough test in both cases I am prepared to say that I do not believe the ray possesses any therapeutic properties, and that the alleged actions on germ diseases are only the result of the electrical current and other natural causes. I believe that any action that may reside in the ray is at our command in the well established currents of electricity.

Case No. 2.

Patient came into my office with a fracture of the humerus. The diagnosis was simple. I set these bones, applied splints, then examined with the ray to see if I had secured the bones in proper position. To my surprise I found the bones overlapping at least one and one-half inches. With the assistance of two negro men I made steady traction for several minutes, tightened my bandages, with the satisfaction that the bones were in good position. I have frequently examined this arm to find an absence of the usually large callus always found in fractures that were improperly set. I have set three fractures by this method; have examined them afterward and found no callus or enlargement at point of fracture. I have examined five fractures set by the usual method. In every case I found a large callus, which always interferes with the functions of the surrounding soft parts. In two cases of fractures of the tibia I found the bones overlapped at least one and one-half inches, in two cases of fracture of the humerus, I found the same condition, and in both cases a very large callus. I am satisfied that many cases of supposed injury to the nerves which develop late is the result of the pressure of a large callus so essential to union in overlapped bones. The value of the X-ray in office work is conspicuous in the detection of both fractures and dislocation, in the demonstration of integrity wherein fractures and dislocation do not exist, in the detection and localization of foreign bodies, in the diagnosis of growth composed wholly or in part of bone.

In recent fractures the exact position of the fragments before and after reduction can be made out. With the aid of an assistant a faulty adjustment may be rectified at once, displacements of the fragments may be discovered long before the time for reapplication of splints.

In old fractures the information to be gained by the X-ray is invaluable. Only in this way can we determine the position of the fragments and the point of impingment, and the cause of impaired function. There are many cases in which the fluoroscope gives vastly more important information than the photograph, because one sees in succession the parts from every point of view. Dislocations, old or new, can never be overlooked in fluoroscopy. In uninuted fractures the situation of the bone is detected with ease. I recall one instance when the physician blamed himself and the patient—the physician for non-union of the humerus when a fluoroscopic examination showed the bones in correct position. Other not infrequent uses of the rays in general surgical work are the demonstration of bone diseases, exostoses, tumors, loss of substance, irregularities in contour cases, variations in size.

In one instance of an osteosarcoma, I detected the growth in its early stage. In tumors of bony origin, deduction based upon density are easily made.

Irregularities in outline from erosions, necrosis, bony deposits and all forms of bone disease attended by change of form, widen still further the scope of X-ray usefulness.

The following cases will serve to illustrate the advantage of the ray in examining soft parts for foreign bodies :

Case 1. Boy, age ten years, while out playing received from a parlor rifle a bullet wound of the belly. A physician was called in and diagnosed a serious condition, and explained to his parents what had probably happened to the intestines, as the bullet passed through the abdominal cavity. A consultation was suggested and another surgeon called in, and made the usual prognosis that if the little fellow lived three days he had a fighting chance. A few months later I examined this case before the ray, and found the ball safely resting in the muscles of the abdominal walls. It had passed obliquely about two inches from point of entrance.

Case 4. Young lady, age thirty, with discharging sinus to the left and on a level of the second lumbar vertebra. Three years ago this case was operated on by Dr. Willis Westmoreland for stone in kidneys. He cut for the left kidney, but failed to find it. He then traced the sinus across in front of the spine to the opposite side. A

few weeks later I examined this case before the X-ray and found a large horseshoe-shaped kidney on right side with a small stone in its substance, with no shadow of a kidney on the left. This case, interesting from its very rarity and in that this patient had taken ether three different times for forty minutes each, with only one kidney, and it diseased.

Case No. 6. Young man received a bullet wound of the thigh, which entered on the lower outer side, struck the femur, glanced upward and inward and came within two inches of the surface on the inner side. The wound was never probed, but continued to ache and throb. On the tenth day I examined with the X-ray, located the bullet twelve inches and on the opposite side from point of entrance. I placed patient on table and with cocaine made an incision a little wider than the width of my scalpel and removed the ball. In a short time the wound healed.

Two weeks later patient came into my office complaining of a throbbing pain which seemed deeply seated, with slight elevation of temperature. I examined him with the ray and found a small spicula of detached bone, with a rough, uneven surface immediately surrounding it; guided by the X-ray I introduced the needle and withdrew considerable pus. Through this needle I injected one-half ounce carbolic acid, withdrew my needle. All symptoms subsided and I have frequently examined this patient to find no evidence of bone disease other than elevation of the united spicula.

